# “Is the health system ready?” A qualitative exploration of stakeholders’ opinions about the feasibility of preconception care services in the Nigerian health system

**DOI:** 10.1186/s12978-022-01454-2

**Published:** 2022-06-29

**Authors:** Oludoyinmola O. Ojifinni, Latifat Ibisomi

**Affiliations:** 1 School of Clinical Medicine, Faculty of Health Sciences, University of the Witwatersrand, Johannesburg, South Africa; 2 Division of Epidemiology and Biostatistics, School of Public Health, University of the Witwatersrand, Johannesburg, South Africa; 3 Nigerian Institute of Medical Research, Lagos, Nigeria

**Keywords:** Preconception care service deployment, Health system readiness, Preconception care policy, Integration of preconception care service, Collaboration for preconception care

## Abstract

**Background:**

Preconception care (PCC) services aim to improve reproductive health outcomes through the provision of biomedical, behavioural and social health interventions to women and couples before conception occurs. Countries that have deployed PCC services have policies that guide the services provided. In Nigeria, PCC is poorly developed and is often provided in an opportunistic manner with no guidelines in place to direct the provision. This study explored the opinions of policymakers and health workers about the feasibility of deploying PCC services in the country.

**Methods:**

This study was a qualitative exploration of opinions about PCC service deployment within the Nigerian health system in which 39 in-depth interviews were conducted with policymakers at the federal and state tiers of government as well as health workers at the tertiary, secondary and primary levels of health care. The transcripts were analysed thematically using a hybrid of deductive and inductive coding on MAXQDA 2018 qualitative data analysis software.

**Results:**

Four main themes emerged from the data—issues around policy for PCC, service integration and collaboration, health system readiness and challenges to PCC service deployment. While noting that the country has no PCC policy, participants identified existing policies into which PCC can be integrated. The participants also described the importance of policy to PCC provision and provided information on existing collaborations that can help the policy development and implementation process. Although many of the participants believed the health system is prepared for PCC deployment, they identified challenges related to policy formulation and implementation, including financial challenges that could hinder the process.

**Conclusion:**

Deployment of PCC services in the Nigerian health system is achievable as there are existing health-related policies into which the guidelines can be integrated. However, there is a need to consider the possible implementation challenges and address them as part of the planning process.

## Plain Language Summary

Optimising the health of women and men in preparation for childbearing can influence pregnancy outcomes positively. This optimisation can be achieved through the provision of preconception care. In many low- and middle-income countries including Nigeria, preconception care is provided in a haphazard manner with no guidelines to structure the service. In this article, the findings from discussions with health care providers and policymakers in Nigeria are presented. These findings show that preconception care can be deployed in a structured manner through collaboration between health care providers and integration with the existing maternal and child health services. Guiding policies can be provided by inclusion of preconception care in the existing health-related policies in the country.

## Introduction

Preconception care services have been initiated in many high-income countries to address unplanned pregnancies and poor maternal and child health outcomes [1]. Preconception care (PCC) is the provision of biomedical, behavioural and social health interventions to women and couples before conception occurs [2]. PCC provides an opportunity to provide routine healthcare for women across their lifespan, and this can be integrated with their reproductive life plan [3]. The aim of PCC is ultimately to improve maternal and child health outcomes. To achieve this aim, countries globally have adopted different models of PCC services. A common thread across the various countries that have PCC services is the use of specific national guidelines and policies. National guidelines and policies are important to ensure evidence-informed practice, provide guidance for comprehensive and consistent PCC service provision and prevent confusion of roles [3].

In the United States of America, Canada, Belgium, Sweden, Italy, United Kingdom, Denmark, Hungary, Netherlands, Hong Kong, China, South Korea and Malaysia there are national PCC guidelines for health providers to standardize PCC services for couples [1, 3–5]. These guidelines focus on the use of folic acid in the preconception period, tobacco cessation, alcohol reduction, and management of chronic illnesses [1, 5–7]. In addition, the United States guidelines include reproductive planning and PCC as part of every contact of women of reproductive age with the health system [8, 9]. The packages of care in Sweden, South Korea, Netherlands and Hong Kong include population based methods for raising awareness among the general population to trigger demand [1, 6]. Other models of delivery of PCC services are premarital health check in China which includes medical consultation, risk evaluation, physical examination, laboratory tests and preconception health education [10, 11]. Provision of PCC within primary health care settings by midwives in Sweden includes risk assessment, folic acid supplementation, encouraging healthy diet of fruits and vegetables, counselling on smoking cessation, alcohol reduction and weight management. Individuals with complicated cases are referred to physicians [1]. Provision of PCC by family physicians in Netherlands involves individual consultations with risk assessment and delivery of interventions for identified risks [12]. Although there is heterogeneity across the policies and guidelines in these countries, the availability of guidelines provides structure for the integration of PCC within each country’s health services, addresses peculiar needs among each population group and encourages demand for the services.

In Nigeria, as in many low- and middle-income countries (LMICs), PCC is poorly developed and is often provided opportunistically with no guidelines or policies in place to direct the services [13, 14]. PCC studies among pregnant women in the country have shown awareness of the service among women of reproductive age ranging from 8.3% in Bayelsa State to 20.6% in Sokoto State, 44.2% in Ebonyi State, 63.5% in Osun State and 76% in Lagos [15–19]. In these studies, the proportion of women who accessed PCC before pregnancy was even lower and the most common PCC services provided were folic acid supplementation and dietary modification. However, given the increasing number of women with pre-existing medical conditions such as diabetes, hypertension and sickle cell disease and the higher risk of negative pregnancy outcomes among such women in the country [20–23] there is the need for PCC deployment and guideline development. The guideline should specify who requires the service, when, where and how it should be offered and what components should be provided. This study explored the opinions of stakeholders—policymakers and health workers—about the feasibility of deploying PCC services in the country.

## Methodology

This study was a cross-sectional exploration of opinions about PCC service deployment within the Nigerian health system. The study explored the health system at the tertiary, secondary and primary health care levels using in-depth interviews to obtain the perspective of health workers and policymakers.

### Study setting

Politically, Nigeria operates a three-tier political system with a democratically elected federal government at the national level, state governments in the 36 states and the Federal Capital Territory, each of which is subdivided into local government areas (LGAs) managed by local government authorities [21, 24]. Within the Nigerian health system, the local government authority manages the development, operation and provision of PHC services under the guidance of the National Primary Health Care Development Agency (NPHCDA) [24, 25]. The state governments perform a technical role—training staff, overseeing the activities at the local government level and providing secondary health services while the federal government provides strategic oversight and manages the tertiary health services [21, 24]. This study used multiple sites across the three tiers of government and the three levels in the health system. For the health system aspect of the study, Oyo State was purposively selected for two main reasons. First, it is one of the urban southern states with good access to health services and better reproductive health indices [21, 26]. Secondly, it has tertiary, secondary, and primary health facilities located in one of its LGAs.

### Study population

The study population included policymakers at the federal and state levels and health workers at the primary, secondary and tertiary levels of care. Using purposive sampling, participants were recruited into the study based on their experience and ability to provide information on maternal and child health issues in the country and within the health system. At the federal and state levels, 13 policymakers were selected from Ministries, and Agencies with links to maternal and child health services. These were the Ministries of Health, Education, Sports & Youth Development, Women Affairs, and the Primary Health Care Development Agency at both federal and state levels. The health workers were purposively selected on the premise of their involvement in maternal and child health services. Working with the varying staff population at each of the three levels of health care, three (3) health workers were selected at the primary level, five (5) at the secondary level and 18 at the tertiary level. All the policymakers and health workers approached for the study agreed to participate.

### Data collection

Interview guides containing open-ended questions were developed for the study using information from existing PCC literature. The interview guides were pretested and changes made to ambiguous questions before the study began. The main interview questions for the health workers were: What role is there for preconception care services in your practice? How would you go about integrating preconception care services into your practice? What challenges do you foresee that may affect integrating preconception care into your practice? For the policymakers, the main questions were: How feasible is integration of a formal preconception care service into the existing maternal and child health care services? What opportunities exist for integrating preconception care service into the existing maternal and child health services? What policy opportunities or gaps can you identify as likely to catalyse the integration of preconception care into existing services? What challenges do you anticipate? The first author was responsible for the data collection and conducted all the policymaker interviews. There was no prior interaction between the interviewer and the participants besides the contact made to set up the interviews. However, because she is a Community Physician who has worked with many of the health workers previously, four research assistants were recruited for the health worker interviews. The research assistants were Masters students from the Faculty of Public Health, University of Ibadan, Nigeria who were experienced in qualitative data collection. Being of lower qualification and younger ages than most of the health workers could have affected the research assistants’ ability to probe properly during the interviews. The effect of this was minimised by having debriefing sessions to review each interview and field notes with the first author after each interview. Notes were made on issues that could have been probed further and these were included in subsequent interviews. All the interviews were conducted face to face in the participants' offices and lasted about 30 to 45 min each.

### Data management and analysis

The interviews were recorded with a digital recorder and transcribed verbatim by the research assistants. The first author read all the transcripts and integrated them with the field notes and reflective diaries, comparing them with the audio recordings to ensure there was no missing information. The transcripts were returned to the participants for review and corrections or additions were made as requested. The transcripts were imported into MAXQDA 2018 qualitative analysis software. Thematic analysis using a hybrid of deductive and inductive coding was done [27, 28]. As part of the measures to ensure trustworthiness of the analysis, two independent coders who are not authors on this paper but are experienced in qualitative analysis developed codes deductively by identifying recurrent patterns in four of the transcripts. The first author also coded the same set of transcripts and agreement on final codes was reached during a discussion session by the three coders. The initial set of 25 codes were merged into four main themes, two of which had subthemes (Table 2).

### Ethical considerations

All the study participants were provided with information sheets giving details of the study and consent was obtained for both the interviews and the recording. No identifying information was obtained; the transcripts were de-identified and stored in a password protected computer accessible only to the authors. Ethical approval for the study was obtained from the ethics committee of the University College Hospital (UCH), Ibadan, Nigeria, the Oyo State Ministry of Health, the Federal Ministry of Health, Nigeria and University of Witwatersrand Human Research Ethics Committee, Johannesburg, South Africa.

## Results

Thirty-nine transcripts were analysed in this study—there were 13 policymaker transcripts (six at the federal and seven at the state level) and 26 health worker transcripts (13 specialist physicians and five nurses at the tertiary health care level; three specialists and two nurses at the secondary health care level; one specialist and two nurses at the primary health care level). The specialties covered in the study were those relevant to the management of pre-existing medical conditions that require PCC. They  included: Cardiology, Community Medicine, Clinical Nursing, Endocrinology, Family Medicine, Haematology, Nephrology, Neurology, Obstetrics/Gynaecology (Ob/Gyn), Paediatrics, Psychiatry and Public Health Nursing. The health workers had been in service for between one and 32 years while the policymakers had worked for between four and 30 years. The age and sex distribution of the participants is shown in Table 1.

**Table 1:** Participants' age and sex distribution

	Doctors	Nurses	Policymakers	Total (N = 39)
Sex				
Male	13	0	2	15
Female	4	9	11	24
Age				
26–35	1	2	1	4
36–45	9	5	3	17
≥ 46	7	3	9	19

### Study themes

The themes and subthemes identified from the data are summarised in Table 2.

**Table 2:** Themes identified in the study

Theme	Subtheme	Description
Issues around policy for PCC	Policies related to PCC	While the participants stated that there was no PCC policy, they identified existing policies that could be adapted to fit PCC
	Importance of policy to the provision of PCC	Participants’ description of the importance of policy to driving implementation of PCC
	Collaboration for policy development and implementation	Highlights given on the existing collaborations that could drive PCC policy development and implementation
Service integration and collaboration	–	Description of existing inter-specialty collaborations and referral systems that can drive PCC
Health system readiness	–	Opinions on the readiness of the health system to deploy PCC services
Challenges to PCC deployment	Challenges to policy formulation	Highlights of the possible challenges to developing PCC policy
	Challenges to service provision	Potential challenges to the provision and uptake of PCC services
	Financial challenges	Description of challenges related to funding of PCC services including budgetary allocation that can affect policy implementation and issues around payment for service by potential clients

#### Issues around policy for PCC

Three subthemes were identified: policies related to PCC, importance of policy to provision of PCC and collaboration for policy development and implementation.

##### Policies related to PCC

At both the national and state levels, none of the policy makers was aware of any existing policies that covered PCC. However, they identified policies which could be expanded to include PCC because of their related content. These included the gender policy, national health policy, child health policy, adolescent health policy, national youth policy and the reproductive health policy.

*Although the national health policy covers a range of awareness and sensitization to communities, it does not cover preconception care. We have the national gender policy and the child health policy, but I don’t think they cover preconception care. But they should. There are plans to review the national child health policy and I think the idea of preconception care can be included then*. – **National Level Policy Maker 4**

*I'm not aware of any policy that includes preconception care directly. But there are policies on adolescent health. I'm thinking of the RMNCAH strategy, in the past it was just integrated maternal, newborn and child health but now we have reproductive, maternal, newborn, child and adolescent health strategy (RMNCAH). It is wider, covering the women of reproductive ages which includes the group of people who need preconception care. That is one strategy that can be used or developed to form a guideline for preconception health care services.* – **National Level Policy Maker 6**

*I don’t have comprehensive knowledge about the policies, but none of the few that I've worked with covers preconception care. But I think it will be appropriate for it to be in the reproductive health policy.* – **State Level Policy Maker 6**

Some of the participants opined that implementation of some programs and interventions in the existing health related policies provide an opportunity for PCC. However, they stated the need to have specific guidelines to structure PCC service delivery.

*I think preconception care is already in our national health policy because prevention of maternal mortality is part of the national health policy. How will we do that if we don’t do preconception health care? Not exactly as you described it but embedded in those programs and activities guided by the health policies, we include preconception care. The Federal Ministry of Health has also realized that there is a gap, and we need to have structured guidelines, but we have not started yet.* – **National Level Policy Maker 2**

*The Family Life and HIV Education curriculum covers preconception care, because it is giving sexual and reproductive health information inbuilt with life skills to young people, which is part of preconception care. So already, it is integrated in little bits into the different subjects that the students are taught.* – **National Level Policy Maker 3**

*I know the National Youth Policy is relevant in this case, our focus is ensuring the wellbeing of young people. And we have a National Strategy for Adolescents and Young People … it captures the knowledge gap but there isn't any policy that speaks to preconception care directly for now*. – **National Level Policy Maker 5**

##### Importance of policy to the provision of PCC

The policy makers at both the national and state levels described the importance of policy to delineate roles and responsibilities in the provision of PCC services. In their opinion, implementation of PCC services can only be properly driven when there is a well-known policy providing direction to all stakeholders.

*The federal ministry of health must make the policy to drive the process, informing the government at the lower levels and giving direction on how to provide the services. Once there is a policy, there will be direction because policy leads to coordination which gives direction on what to do during implementation.* – **National Level Policy Maker 3**

*Some private health facilities may be able to offer preconception care services, but as you know, their services are for profit. The government must include rules in the policy for any private hospital that wants to offer such services to have basic infrastructures and personnel that can provide the services. The policy also needs to state details of the services and limits that the facilities cannot go beyond to avoid complications.* – **State Level Policy Maker 2**

The health workers also weighed in on the need to have a policy to entrench PCC into the existing primary health care system. Describing the need to improve political will at all levels of government, they spoke about the importance of policy and guidelines like what exists for other health programs such as antenatal care.

*Politicians often make promises which they end up not fulfilling, you know... They introduce a program today and tomorrow it is cancelled. The ministry of health, the primary health care board, the federal government all have to be involved for preconception care to work. It cannot be done at the grass root; nothing can be done unless it is taken up by the government. Then they can make it compulsory with a solid program inculcated in the primary health care service. There needs to be something compelling it like we have for antenatal care because this program needs to come before antenatal. That is when it will be able to stand*. – **Primary Care Level Clinical Nurse**

While describing the importance of guidelines and policies, one of the tertiary care level obstetricians spoke of an ongoing process where guidelines for the management of pre-existing medical conditions are being prepared. This he believed should also cater for preconception care services.

*There is no doubt that we should have that policy, but for now we are targeting pregnancy outcomes … we are doing guidelines for medical conditions which can also be preconception care. We are at the verge of doing guidelines for hypertension in pregnancy, other conditions like anaemia in pregnancy, and some other medical conditions in pregnancy. And hopefully we are going to have preconception components of those guidelines*. – **Tertiary Care Level Ob/Gyn 1**

This statement is in tandem with that of one of the national level policy makers who stated that the need for PCC guidelines had been identified at the national level and steps were being taken to provide a guiding policy. This policy will guide implementation and is expected to direct the integration of PCC into existing programs.

*… We are like the thermometer of the Nigerian people when it comes to issues relating to making policy on their health. We have seen it, our thermometer has picked it and we are already acting, taking necessary steps that will lead to the formulation of the policy. And we don’t formulate policy without developing the implementation strategy. We mobilize relevant stakeholders that will do the situation analysis and produce effective strategies for implementation of the policy. And thank God it is not going to entail the formation of new structures. We have existing structures that we will just integrate the program into.* – **National Level Policy Maker 2**

##### Collaboration for policy development and implementation

As part of the discussion on the process of policy development, the policy makers spoke about the opportunities for integration of PCC into the health-related policies in existing collaborations between ministries. They noted that taking advantage of these collaborations, relevant stakeholders can be identified, and a special team created to work on PCC without being stalled by the bureaucracies of the civil service.

*We used to have collaborative efforts between the Ministry of Women Affairs, Ministry of Health, Ministry of Education and Ministry of Information. We call them line ministries because the mandate of one affects the other. There is a maternal and child health policy that was developed by health in collaboration with some stakeholders from the line ministries. So, this one also can be done together, jointly. They can all include preconception care in their sensitization and advocacy programmes.* – **National Level Policy Maker 4**

*We have what we call line ministries, just like a joint program. These line ministries can set up a special committee. For instance, on HIV we have a Critical Mass Committee, and that has been driving interventions in line of HIV. It's not about following normal civil service routes, you know, that delays a lot of things. But when you have a critical team, you know this is the team, they work as if they are separate from the organisation … but the Ministry of Health should coordinate in collaboration with other line ministries.* – **National Level Policy Maker 5**

*I don't know who will draft the policy, but I know there is a body that drafts policy, and they should involve experts from related fields. The Ministry of Health is very pertinent and then Women Affairs since this has to do with women … if the relevant bodies are carried along it will help*. – **State Level Policy Maker 4**

#### Service integration and collaboration

The health workers described existing inter-specialty collaborations, teamwork and referral systems which are platforms through which PCC services can be incorporated into the health system. Further, they highlighted the possible roles of medical associations and health workers in training staff for the deployment of PCC services.

*… What we normally do is we manage the cardiac problem, and the obstetrics doctors manage their own aspect until delivery. Most of the patients that we get here are referred patients. Young people can also come for routine screening, check their blood pressure, blood sugar, ECG, some of those basic tests to see if there are things that can cause problems in future. That will need a special preconception clinic or screening in the general outpatient clinics. Once they detect any abnormality, they can refer, and we will manage if it is related to our own area. I know that the endocrinology unit runs a clinic for the pregnant women who have diabetes. The management is joint, with the obstetric unit. The same thing for people with HIV, we have done this several times.* – **Tertiary Care Level Cardiologist**

*I expect that whatever government or policy makers are going to do, they would not do it in isolation without the medical association. For instance, in training and providing facilities; and then they call something training of trainers. So, we can train a set of people who can now go further and train other people.* – **Tertiary Care Level Ob/Gyn 1**

#### Health system readiness

Regarding the state of readiness of the health system for the deployment of PCC services, the participants' opinions differed. Among the policy makers, some believed that the health system in its current state is prepared and even needs PCC services to address issues like unwanted pregnancy and pre-existing health issues. Others however stated that the health system is not fully prepared particularly with respect to funds and human resources but as policies are developed and or expanded, the state of readiness should improve.

*The health system will be ready, don’t worry. We’re working on all our policies. Like I said prevention, prevention, prevention is the way to go. That is the way out, prevention is the way to go and we’re working towards it.* – **National Level Policy Maker 1**

*The society will be ready for it, because every young girl and every young man wants to have a child. If there are some issues that are there that they need to identify early, it* [PCC] *will help.* – **National Level Policy Maker 3**

*Yes, the health system is very ready. The only thing is that the health system may not have sufficient funds and then of course human resources may not be as much, but we are ready.* – **National Level Policy Maker 6**

*I think the health system is ready because of the issue of unwanted pregnancies. If something like preconception care is available, I think it will prevent it a little bit.* – **State Level Policy Maker 7**

On the part of the health workers, the participants' opinions also differed. Most believed the health system is not in the right state to accommodate a new program like PCC. They cited the current challenges of inadequate personnel and resources for basic health care services. Others believed that the available resources are adequate and PCC service deployment would not be expensive and can be covered within the existing health framework.

*The health system* (laughing) *I can say is already overwhelmed with inadequate funding, brain drain, the existing programs … but if we are positive about it, we can attain it. If the political will is there from our government, we can achieve it.* – **Tertiary Care Level Clinical Nurse Ob/Gyn**

*As at now I am not sure because you know if we say the health system, too many things are involved. We have the personnel, (they are the doctors and the nurses), infrastructure also you have the government policy and then the funding is there. So, I can only talk for myself as a doctor because I know that I am ready, and I know that my colleagues are ready too. But then, the available infrastructures are not enough for the O & G care. Then of course in our environment preconception care will be good but I think there are other pressing demands. So, I am not sure right now that our health system is ready for such an integration process.* – **Secondary Care Level Ob/Gyn**

*As things are now, I will say no. … We are struggling with other more rudimentary issues in providing health care to begin to talk about high level things like some of the services included in preconception care. Fine, of course the counselling can go on. There is not much required for that, but some of the other services like the testing and the treatments are things we are not prepared for at this time. There are still issues with other more basic things like providing universal health coverage, universal health insurance. Those are more immediate concerns. It appears that we are not prepared to take on all the screenings, testing and maybe treatment as part of preconception care for now.* – **Tertiary Care Level Neurologist**

*Well, I don't think so because people are finding it difficult to support basic things like malaria, hypertension, diabetes, they are struggling. The health system is not able to accommodate everybody because of dwindling funds, attitude of workers. So, preconception care like I told you is not a high priority now.* – **Tertiary Care Level Ob/Gyn 2**

*Would the health system be ever ready for anything? We have to make ourselves ready. The structure, the personnel, the professionals, and specialists that are needed should be there and of course, money should be there. If not, the policy makers will say they are not ready, those who are going to carry out the services too will say policy makers have not released funds, it becomes a vicious cycle.* – **Tertiary Care Level Public Health Nurse**

*… Yes, the health system is ready. Why do I say it’s ready? We have everything it takes. We have the manpower, the equipment it takes to do all these things they are always with us, they are available; in fact, it is not particularly expensive. So yes, we are ready. It’s not going to be changing anything much, just that we need to go out, that’s all.* – **Tertiary Care Level Paediatric Cardiologist**

#### Challenges to PCC deployment

Under this theme, participants identified challenges to policy formulation and implementation, service provision and financial challenges.

##### Challenges to policy formulation

Timeline for policy review was identified as a possible challenge to the integration of PCC into existing policies. Some of the policy makers stated that there were health-related policies that had been reviewed shortly before the time of the study and reviews are only done once in four to five years. They noted that the review cycle may be affected by change in government particularly when the program is not in line with the new government’s priorities. However, one of the participants suggested the development of short-term plans and strategies in the interim while anticipating full inclusion at the next policy review.

*Well, policies and strategies are revised every four years. The last revision of the Adolescent Health policy was about a year ago. So, we have to wait for four years before any other revision which means that’s when preconception care can be included. And if there is a change in government, it can reduce the chances because another leader may not believe in the strategy and feel that it’s not a priority. So, leadership is key.* – **National Level Policy Maker 6**

*Unfortunately, we just finished the review of the National Youth Policy …. However, there is no crime in developing pre-policy strategy … we can develop a strategy from the policy. … We can have a preconception strategy for specific groups of people, just have a 2-year plan, 3-year plan.* – **National Level Policy Maker 5**

Some of the policy makers expressed concerns over the appropriateness of inclusion of PCC in the FLHE curriculum. In their opinion, provision of PCC services at secondary school level may be seen as promoting sexual activity and thus lead to an increase in unwanted pregnancies which may be counterproductive.

*Now we are saying they should incorporate it* [PCC] *into the FLHE curriculum. My fear is that it may be abused and seen as encouraging unwanted pregnancy among the secondary school students. All these reproductive things are not encouraged for that age. I think reproductive planning is for those who are married.* – **State Level Policy Maker 7**

##### Challenges to service provision

The possible lack of equipment, infrastructure and adequate skills mix particularly at the PHC level was identified as likely to hinder deployment of PCC services. Mention of the PHC level was because it is at the grassroots level – closest to the community – where basic health care, including PCC should be available. However, the lack of an adequate skills mix due to absence of an enabling environment for health personnel hampers staff retention at the PHC level and makes the workload for the existing staff excessive. Furthermore, the possibility of misunderstanding the motives of PCC was highlighted and the need for improved awareness at population level to address this was mentioned.

*One of the challenges is the availability of equipment and facilities. For instance, are there equipment for diagnosing sickle cell at the PHC level? Basic genotype without giving inconclusive results is something that should be done at that level. There is also the issue of additional workload for the nurse/midwives, health educators and others at the PHC level. Although it enriches the system which is a merit because of the preventive intervention that will reduce the workload of curative care. However, it may be seen as additional work for the health workers.* – **National Level Policy Maker 1**

*We would need to mobilize resources from every area, not just financial. We need technical support and the support of the beneficiaries of the service. Work must be done on health promotion, communicating the objectives of the program to the Nigerian public so that it will not be mistaken like they took polio vaccine for something else … We also need skills for programming, skills for service delivery, like the skills mix you have in the primary health care. Most of the primary healthcare services are at the grass root level, at the community level but the skilled health workers run away because there are no facilities, social amenities to cater for their family needs. They need schools for their children, they need recreational centres around them, they need good roads, potable water, power.* – **National Level Policy Maker 2**

The health workers also stated the need for staff training and improved awareness at the community level to ensure provision and uptake of the services.

*Like I tell people policies are made. Nigeria has policies and guidelines. So many people have sat down with so many of these things … and drawn up policies. We need to go back and train people, the workers and of course make the people aware that such services are available at the community level*. – **Tertiary Level Ob/Gyn 1**

One main challenge cited by the health workers is the fact that pregnancies are often not planned. This and the fact that the proportion who use the existing maternal health services is low, they believed may affect uptake of PCC services.

*I think the biggest challenge is the fact that most pregnancies in this part of the world are not planned. It takes planning for a pregnancy to realise the need for preconception care. But most people don’t plan pregnancy like that. Pregnancy just happens and by the time they realise, the time for preconception care has passed. Besides that, uptake of maternal services is low. Up to 60% of women who claim to use antenatal care don’t even see health workers, they see traditional birth attendants which is not optimal. We should improve that.* – **Secondary Level Ob/Gyn**

##### Financial challenges

Describing the importance of ensuring an implementation strategy to drive the deployment of PCC in the country, the policy makers stated the need for budgetary allocations to ensure financial provisions to back the PCC policy when it is available.

*Well sometimes the problem is not with policy, but with the implementation. There is often no accompanying implementation strategy or enabling environment for the policy to thrive. The government itself should realize the importance of this concept and put mechanisms on ground to make it thrive. There should be budgetary provision to drive the process. All these take time; anything that has to do with policy formulation, implementation will be gradual*. – **State Level Policy Maker 6**

*… With the new policy of government now, money that is meant for a specific activity cannot be moved to another activity. Everything must be covered in the budget before you can do it. It must be integrated into the budget first for you to have funding implementation*. – **National Level Policy Maker 4**

*Another thing that could hinder it will be poor implementation of the strategy due to lack of funds. … If there is no budget line for those activities, then that will definitely hinder implementation, you just have it on paper there is nothing to start with*. – **National Level Policy Maker 6**

Financial challenges including the cost of establishing the service were described. This according to the study participants would be the cost of formulating a guiding policy and the cost of deploying the service – staff recruitment and training and promoting the program to ensure uptake.

*Nigeria as at now doesn’t have a preconception care policy and to formulate the policy, we need funds, we need time. Funds to train the appropriate cadres, put some structures, drugs and consumables in place for preconception care, publicity, and health education. Money is the baseline for everything*. – **Tertiary Level Public Health Physician 2**

*The main challenge is finance* – *for the media to disseminate information, production of handbills and posters. Besides, the state is short staffed; some local government areas have only one nurse covering the whole area. There needs to be political will so the government can provide the needed funding for all these.* – **State Level Policy Maker 1**

Other potential costs highlighted are the cost to clients. The participants wondered if the potential cost of the service to the clients would not discourage uptake.

*And the socioeconomic impact on the women. What I mean is that is it going to be an extra burden to them? Is it going to be free? That may also affect implementation* – *the financial aspect*. – **National Level Policy Maker 6**

*Let me just say one final thing, financial implication of preconception care to these persons, who takes care of it? Will the tests be free to encourage people to come for preconception care? When you tell a random person on the street to go for preconception care, who takes up the responsibility of paying for the investigation?* – **Tertiary Level Endocrinologist**

## Discussion

Although existing literature shows that PCC services are provided in Nigeria, the services are poorly developed and not guided by any in-country standard operating procedure [13, 29]. This study is novel in that it explored the opinions of policy makers and health care workers about the feasibility of structured preconception care (PCC) services in Nigeria which has not been done previously. Previous PCC studies have assessed knowledge, awareness, and utilisation of PCC services among health workers and women of reproductive age in the country [13, 16–19, 30]. The findings in this study highlight existing opportunities for policy development and implementation, describe issues related to health system readiness for provision of services and identify possible challenges at policy and health system levels.

While acknowledging the absence of a PCC policy in the country, the study participants highlighted the importance of having a policy and emphasised on the integration of PCC services into the existing health system. Both the policy makers and the health workers believed that a policy would provide a framework for proper implementation of PCC services. In addition, the policy makers identified some health-related policies into which PCC can be integrated. They also described existing collaborations between the government agencies that could be used to hasten the policy development process. Among the policies mentioned in the study are the reproductive health policy, adolescent health policy and the national health policy. A review of the health-related national policies in Nigeria shows that there are targets for various aspects of maternal and child health, but not for PCC. The National Reproductive Health Policy and Strategy to Achieve Quality Reproductive and Sexual Health for all Nigerians places emphasis on preventive and promotive measures for the reduction of maternal, perinatal, and neonatal morbidity and mortality. These measures are integrated with treatment and rehabilitation in a multi-disciplinary and multi-sectoral manner [31]. Embedded in the 2016 National Health Policy is the Reproductive, Maternal, Neonatal, Child and Adolescent Health (RMNCAH) goal [21]. This RMNCAH goal aims to integrate RMNCAH services into the continuum of care, reduce morbidity and mortality and promote universal access to comprehensive sexual and reproductive health services for adolescents and adults throughout the life cycle. In addition, as part of initiatives to combat non-communicable diseases (NCDs) in the country, the National Health Policy aims to institute universal screening and genetic counselling for the populace [21]. Although PCC is not specifically stated, the framing of the RMNCAH goal and the plan to control NCDs intuitively provides room for adaptation and integration of PCC services. The Global Action Report on Preterm Birth recognises PCC as the weak link in the continuum of care and states the importance of the preconception period for improving the health and wellbeing of girls and women, as well as those of boys and men [32]. If achieved, the integration of PCC into the continuum of care in the National Health Policy will address this weak link and aid progress towards improving health and wellbeing in adolescence and adulthood.

The National Adolescent Health Policy emphasises responsible sexual behaviour and positive attitudes to sexuality as a means of preventing unwanted pregnancy. The policy aims to facilitate the provision of effective, accessible information, guidance and services for the promotion of health and prevention of health problems through integration of these services into the school curricula and service provision in areas where adolescents can be found [31]. This relates to the Family Life and HIV Education (FLHE) curriculum mentioned by the study participants. One of the objectives of the FLHE curriculum is to provide information and skills necessary for individuals to make rational decisions about their sexual health [33]. The curriculum identifies six themes (human development; personal skills; sexual health; relationships; sexual behaviour; society and culture) which are incorporated into the different school subjects at the secondary school level [33]. Various aspects of PCC can be integrated into these themes. For example, genetic issues may be absorbed into human development while reproductive life planning can be integrated into sexual health and sexual behaviour. Only the national guidelines for the prevention of mother-to-child transmission of HIV (PMTCT) includes preconception care as a primary prevention strategy among women infected with HIV [34]. What is missing is stating the mode of implementation of PCC as a prevention strategy and indicators for evaluation within the policy.

For PCC to be properly implemented in the country, there is a need to integrate the services within the existing health care system. The health workers in this study described collaborations between different specialities which can provide a foundation for integration of PCC. The Nigerian PHC system promotes the PHC-under-one-roof model where all services required by an individual are expected to be available at a single visit without any need for special clinics [25, 35]. Two-way referrals between the primary, secondary and tertiary care levels direct patients from higher to lower levels and vice versa depending on the need [35]. Within the tertiary health care level, referrals may also be needed between specialties. As described by the health workers in this study, the specialties have their methods of referral and collaboration to provide care to patients who require multiple teams to address their health problems. This implies that deploying PCC services in an organised manner will not require developing new systems, although the existing services in the health system may need to be modified.

In terms of readiness for structured PCC services within the health system, the opinions differed among the study participants. Whereas some believed the health system as it exists is ready for deployment of PCC services stating the need to address issues like unintended pregnancy as justification; other participants believed that some adjustments are needed including training of personnel and provision of funds to run the program. However, some of the participants argued that the challenges being faced with health service provision may hamper integration of a new program. In keeping with the opinion that the health system can deploy PCC services, previous studies in Nigeria have shown that PCC services are being provided minimally in the country [13, 17–19, 29, 30]. Further, potential PCC users believe it is important and many are willing to use it if it is available [16, 36]. However, the opinion that the health system is not prepared for deployment of PCC services can be justified by studies among health workers which show that there is a need for increased awareness and training to improve provision of PCC services [13, 30]. In addition, as stated by the health workers in this study, uptake of maternal health services in the country is generally low. There is poor uptake of antenatal care (ANC), delivery, and postnatal care services [24, 37]. For instance, the 2018 Nigeria Demographic and Health Survey (NDHS) showed only 67% of women aged 15–49 years received ANC, 43% had skilled attendance and delivery and 42% had postnatal check [26]. It has been shown however that using PCC services improves the use of ANC and other maternal health services [38]. PCC may therefore be a solution to the problem of poor uptake of maternal and child health services.

In this study, the potential challenges to PCC service deployment in the country were discussed. These challenges ranged from policy formulation to implementation, service provision and finances. The description of the possible challenges may be understood in the context of maternal health services in the country. The services provided at the primary health care (PHC) level include ANC, delivery services and postnatal care. Complicated cases are referred to the secondary and tertiary levels as appropriate. In addition to maternal health services provided within the formal health system, which includes public and private providers, traditional healthcare providers are also involved in maternal healthcare [24]. Access to health services is affected by educational and socioeconomic status and is often dependent on individuals’ ability to pay out of pocket as health insurance systems are not fully developed in the country [24, 39]. In addition, the use of either the formal or traditional providers is often influenced by educational and socioeconomic status. Given this context, the participants' description of the importance of political will and the impact of change in government is understandable. These factors must be considered in the formulation of policies and guidelines for PCC as they will impact on the policy implementation.

## Conclusion

Formal deployment of preconception care (PCC) services in the Nigerian health system is achievable given that there is some level of awareness and some uptake of the available services albeit provided in a haphazard manner. The road to the attainment of this aim may however be tortuous given the challenges currently experienced with provision and uptake of maternal health services in the country. Nevertheless, the foundation for integration of the services has been laid by the phrasing of several health-related policies in the country. The implementation and evaluation structures can also be easily developed using models from countries where the services already exist. There is willingness among the health workers to provide the services as they described existing systems that can be modified to embrace the provision of PCC. With advocacy, it is possible to generate adequate political will to ensure funding for implementation and uptake of PCC in Nigeria.

## Data Availability

Due to the qualitative nature of the study, the data generated are not publicly available. However, further information about the data is available from the corresponding author upon reasonable request.

## Abbreviations

HIV: Human Immunodeficiency Virus
FLHE: Family Life and HIV Education
IDI: In-Depth Interview
LGA: Local Government Authority
NGO: Non-Governmental Organisation
NPHCDA: National Primary Health Care Development Agency
Ob/Gyn: Obstetrician/Gynaecologist
PHC: Primary Health Centre
MTCT: Mother to child transmission
RMNCAH: Reproductive, Maternal, Neonatal, Child and Adolescent Health
UI/UCH: University of Ibadan/University College Hospital
